# Supplementary material for: Augmenting a ResNet + BiLSTM Deep Learning Model with Clinical Mobility Data Helps Outperform a Heuristic Frequency-Based Model for Walking Bout Segmentation
Source: Sensors (Basel). 2025 Oct 13;25(20):6318. doi: 10.3390/s25206318 (PMC12568077; doi:10.3390/s25206318)
Supplement: Supplementary file 1 [file sensors-25-06318-s001.zip › sensors-3872173-supplementary.pdf]

Supplemental S1

Supplemental 1.1: Description of Datasets

S1.1.1. PAMAP2 Dataset

This open dataset was created as a benchmark dataset for physical activity monitoring (Reiss, 2012). The dataset contained 9 participants (8M, 1F; age: 27.2 ± 3.3 years; height: 179.4 ± 8.4 cm, weight: 80.9 ± 10.3 kg) wearing multiple IMU sensors (Colibri wireless IMUs, Trivisio) sampled at 100 Hz while completing 18 varying activities. For the purposes of developing a clinical gait detection model for this study, the only sensor utilized was the IMU attached on the dominant side ankle (Figure 3.1a). There are 12 activities that were completed during the protocol (lying, sitting, standing, walking, running, cycling, Nordic walking, ironing, vacuuming, jumping rope, ascending and descending stairs) and up to six optional activities (watching TV, computer work, driving a car, folding laundry, and playing soccer). Data from Subject 109 in the dataset only contained labeled data for jumping rope and was therefore excluded. All remaining subjects were included. A total of 13 activity labels were identified in the datasets used and updated during subsequent preprocessing.

S1.1.2. Healthy Lab Dataset

This dataset was collected as part of a concurrently collected study. The McMaster Research Ethics Board approved the study (MREB 6120), and all participants provided their informed consent prior to enrollment. In addition to the primary study, 14 healthy participants (10M, 4F; age: 25.0 ± 4.4 years; height: 180.1 ± 9.1 cm; weight: 77.1 ± 13.4 kg) consented to additionally wearing one sensor (IMeasureU Blue Trident; Vicon Ltd., Oxford, UK), sampled at 1600 Hz, on each leg at the anterior-medial aspect of the proximal tibia and attached using semi-elastic straps (Figure 3.1b), while completing five different activities. The activities included three walking trials (i.e. walking in a straight line, walking with toe-out, and walking in a slalom pattern, all at a self-selected speeds), a static trial, and non-gait ambulation. The static trial included data where the participants were standing or sitting, with limited to no movement. The non-gait ambulation trial simulated slower movements (e.g., sweeping) that might be completed during activities of daily living but should not be considered classified as gait for gait analysis purposes. Following collection, the sensors were removed and downloaded using the CaptureU software (Version 1.3.1) and downsampled to 100 Hz. Data from left and right sensors were concatenated together into a single structure, with non-labeled data removed.

S1.1.3. Clinical Dataset

The clinical dataset was created as a subset (n=32) from a larger study utilizing wearable sensors to longitudinally monitor gait in a cohort of older adults (12M, 20F; age: 65.7 ± 7.8 years; 168.0 ± 9.9 cm; 92.3 ± 23.9 kg) with OA awaiting knee (n=25) or hip arthroplasty (n=7). The Hamilton Integrated

Research Ethics Board (HiREB 16236) approved this study, and all participants provided their informed consent prior to enrollment. Patients wore one sensor (Axivity AX6; UK), sampled at 100 Hz, on each leg at the anterior-medial aspect of the proximal tibia (Figure 3.1c), attached using waterproof medical grade tape (Simpatch Adhesive Patch). Sensors were worn for one week to capture free-living gait and activity data, as well as in-clinic performance-based functional tasks recorded with markerless motion capture (Theia3D, Kingston, ON). For the current study, only quiet standing and a 60-second self-selected walking speed tasks were manually labeled since not every patient completed all functional tasks and represents the minimum dataset across all patients. While markerless motion capture video data is not included in the current study, the data were used to calculate gait speed as described in Outerleys, et al. (Outerleys et al., 2024a). To improve robustness across gait speeds for the final model, patients' gait speeds were classified as "slow" (<0.8 m/s), "average" (0.8-1.2 m/s), and "fast" (> 1.2 m/s) based on previously reported mean gait speeds for OA patients (Marcum et al., 2014; Wiik et al., 2017). Data from left and right sensors were concatenated together to form a single data structure.

Supplemental 1.2: Data Collection Protocols

S1.2.1. Healthy Lab Dataset

As part of a concurrently collected study, 14 healthy subjects (10M, 4F; age: 25.0 ± 4.4 years; height: 180.1 ± 9.1 cm; weight: 77.1 ± 13.4 kg) had gait recorded during three walking trials at a self-selected speed. The primary study collected gait data from another motion capture system, which were not analyzed for the current study. Participants wore one sensor (IMeasureU Blue Trident; Vicon Ltd., Oxford, UK), sampled at 1600 Hz, on each leg at the anterior-medial aspect of the proximal tibia and attached using semi-elastic straps. Following set up and calibration, the following gait tasks were collected on an indoor walking track:

1) 4 x 10 m straight line walk.
2) 2 x 10 m "toe-out" straight line walk.
3) 2 x 10 m slalom walk, where the subjects navigated around cones in a serpentine pattern.

Following the completion of the walking trials, subjects stood or sat down while they waited to begin the final sensor collection, and kept the IMeasureU sensors on the shanks on and recording during this process to collect static and semi-static data, approximately for 2 minutes. Afterwards, subjects completed common activities of daily living that are ambulation but not typical walking, to simulate slower gait that might be completed during activities of daily living but should not be considered classified as gait for gait analysis purposes. These trials consisted of:

1) Picking up 12 cones arranged in a 3x4 grid, spaced approximately one meter apart, in any order they wished.

2) Broom sweeping the area around where the grid had been arranged for 1 minute.

Sensors were removed and downloaded using the CaptureU software (1.3.1) and downsampled to 100 Hz. Data from each sensor were manually labeled using a custom MATLAB script (The Mathworks, Natick, MA), identifying each active section as "walking", "static" or "other", while non-classified data were removed. Data from left and right sensors were concatenated together into a single structure.

S1.2.2. Clinical Dataset

A subset of data from a larger study utilizing wearable sensors to longitudinally monitor gait in a cohort of older adults (12 M, 20 F; age: 65.7 ± 7.8 years) with OA awaiting knee (n=25) or hip arthroplasty (n=7) were included. With respect to gait, OA patients typically walk slower with an atypical gait compared to healthy control subjects, although level of function can vary greatly with pain [13]. Algorithms, both machine learning and heuristic, may not be able to accurately classify gait cycles due, especially when patients have a slower gait speed. Wearable sensor data from the patients' preoperative visit, approximately two weeks before a scheduled surgery, was used. Patients were asked to wear one sensor (Axivity AX6; Vicon Ltd., Oxford, UK), sampled at 100 Hz, on each leg at the anterior-medial aspect of the proximal tibia and attached using waterproof medical grade tape (Simpatch Adhesive Patch) to be worn for one week to capture free-living gait and activity data. Following sensor placement, patients were asked to complete a battery of functional tasks recorded with 10 cameras (Sony RX0-II, 60 Hz, Sony Corporation) for post-processing with markerless motion capture software (Theia3D, Kingston, ON):

1) 30 seconds of quiet standing
2) 60 seconds of self-selected pace walking
3) 30 seconds of faster walking,
4) Five repetition sit-to-stand
5) Two steps up and down.

Following the one-week period, patients mailed the sensors back using provided envelopes. Data were downloaded using the OmGui software (1.0.0.43) and manually labeled using a custom Python script in a similar way as described above for the healthy dataset. For the current study, only quiet standing and 60-second self-selected walking tasks were utilized since not every patient completed all five functional tasks and represents the minimum dataset across all patients. Additionally, to the interest in identifying slower gait, faster walking was not included. The step up and down task, while similar to stair ascent and decent, was ultimately not long enough of a trial to be included for potential classification. Data from left and right sensors were concatenated together to form a single data structure.

Markerless motion capture video data was processed in Theia3D and then processed into gait cycles using Visual 3D. Heel strike events calculate spatiotemporal variables, including gait speed, as described in Outerleys, et

al. To ensure robustness across gait speeds for the final model, patients' gait speeds were classified as "slow" (<0.8 m/s), "average" (1.0 m/s), and "fast" (> 1.2 m/s) based on previous reported mean gait speeds for OA patients.

Supplemental S2

Supplemental S2.1

Data labels were updated across all datasets to be consistent during preprocessing. The full original and updated labels for each are displayed in Table S1.

Table S1. Original and updated labels for each dataset.

Dataset	Original Label	Updated Label
PAMAP2	1 – Lying<br>2 – Sitting<br>3 – Standing	0 – Static
	4 – Walking	1 – Walking
	5 – Running	2 – Running
	6 – Cycling	3 – Cycling
	12 – Going upstairs	4 – Going upstairs
	13 – Going downstairs	5 – Going downstairs
	16 – Vacuum cleaning<br>19 – House cleaning	6 – Other
	7 – Nordic walking<br>17 – Ironing<br>24 – Rope jumping	Removed
Healthy	1 – Straight walking<br>2 – Toe-out walking<br>3 – Serpentine walking	1 – Walking
	4 – Static (sitting and/or standing)	0 – Static
	5 – Cone Task<br>6 - Sweeping	6 – Other
Clinical	1 – Quiet Standing	0 - Static
	2 – Self-selected walking (60 seconds)	1 – Walking
	3 – Fast walking (30 seconds)<br>4 – 5 repetition sit-to-stand.<br>5 – Stairs	Removed
